# New Genetic Loci Associated With Chronic Kidney Disease in an Indigenous Australian Population

**DOI:** 10.3389/fgene.2019.00330

**Published:** 2019-04-16

**Authors:** Russell J. Thomson, Brendan McMorran, Wendy Hoy, Matthew Jose, Lucy Whittock, Tim Thornton, Gaétan Burgio, John Duncan Mathews, Simon Foote

**Affiliations:** ^1^Centre for Research in Mathematics, School of Computing, Engineering and Mathematics, Western Sydney University, Sydney, NSW, Australia; ^2^John Curtin School of Medical Research, Australian National University, Canberra, ACT, Australia; ^3^Centre for Chronic Disease, Faculty of Health, The University of Queensland, Brisbane, QLD, Australia; ^4^Menzies Institute of Medical Research, College of Health and Medicine, University of Tasmania, Hobart, TAS, Australia; ^5^School of Medicine, College of Health and Medicine, University of Tasmania, Hobart, TAS, Australia; ^6^Institute for Marine and Antarctic Studies, College of Sciences and Engineering, University of Tasmania, Hobart, TAS, Australia; ^7^Department of Biostatistics, School of Public Health, University of Washington, Seattle, WA, United States; ^8^Centre for Epidemiology and Biostatistics, Melbourne School of Population and Global Health, The University of Melbourne, Melbourne, VIC, Australia

**Keywords:** chronic kidney disease, genome-wide association study, Australian Aboriginal and Torres Strait Islanders, indigenous genetics, gene–environment interaction, urinary albumin creatinine ratio

## Abstract

The common occurrence of renal disease in Australian Aboriginal populations such as Tiwi Islanders may be determined by environmental and genetic factors. To explore genetic contributions, we performed a genome-wide association study (GWAS) of urinary albumin creatinine ratio (ACR) in a sample of 249 Tiwi individuals with genotype data from a 370K *Affymetrix* single nucleotide polymorphism (SNP) array. A principal component analysis (PCA) of the 249 individual Tiwi cohort and samples from 11 populations included in phase III of the HapMap Project indicated that Tiwi Islanders are a relatively distinct and unique population with no close genetic relationships to the other ethnic groups. After adjusting for age and sex, the proportion of ACR variance explained by the 370K SNPs was estimated to be 37% (using the software GCTA.31; likelihood ratio = 8.06, *p*-value = 0.002). The GWAS identified eight SNPs that were nominally significantly associated with ACR (*p* < 0.0005). A replication study of these SNPs was performed in an independent cohort of 497 individuals on the eight SNPs. Four of these SNPs were significantly associated with ACR in the replication sample (*p* < 0.05), rs4016189 located near the *CRIM1* gene (*p* = 0.000751), rs443816 located in the gene encoding *UGT2B11* (*p* = 0.022), rs6461901 located near the *NFE2L3* gene, and rs1535656 located in the *RAB14* gene. The SNP rs4016189 was still significant after adjusting for multiple testing. A structural equation model (SEM) demonstrated that the rs4016189 SNP was not associated with other phenotypes such as estimated glomerular filtration rate (eGFR), diabetes, and blood pressure.

## Introduction

There is an epidemic of renal disease in the Australian Aboriginal population. In the Northern Territory (NT), diabetes deaths in Aboriginal people are increased up to 15-fold, cardiovascular deaths by three- to sixfold (Hoy, 1996), while rates of treated end-stage renal disease (ESRD) are more than 20 times that of non-Indigenous Australians overall, and more than 60-fold greater in some regions (Spencer et al., 1998). The burden of morbidity is great, costs of hospitalization and ESRD treatment are huge (You et al., 2002) and premature deaths of young and middle-aged adults have incalculable impact on families and communities. Studies in the NT Aboriginal communities of Nauiyu, Wadeye, Borroloola (the Outreach communities), the Tiwi Islands, and Angurugu, showed that the rates of hypertension, albuminuria/proteinuria, and diabetes were high, although they differed by community (Hoy et al., 2006b). Relative to the (mostly non-Indigenous) participants in the nationwide AusDiab study, rates of proteinuria in the first three of these Aboriginal communities were increased 2.3- to 8.7-fold, hypertension was increased 2.7- to 3.6-fold and diabetes was increased 6- to 9.6-fold (Hoy et al., 2006b). Incidence increased markedly with age. In addition, there was pronounced overlap of these conditions that also increased with increasing age.

The causes of this disease epidemic are poorly understood but are very likely linked to changes associated with compressed transition to a quasi-western lifestyle in the last 50 years. However, smoking and obesity do not appear to fully explain the disease (Wang and Hoy, 2003), and central fat deposition, inflammation and infections, psychosocial stress, birthweight, and a genetic propensity to develop renal disease in the presence of other factors have been suggested as causes. For example, streptococcal impetigo, and other markers of Group A Streptococcal infection are predictive of albuminuria in Aboriginal children and adults (Van Buynder et al., 1992; Goodfellow et al., 1999). The same association has been seen in this population (Hoy et al., 2012).

Renal disease in the Australian Aboriginal population has classic pathological findings, characterized by the presence of fewer, larger glomeruli with or without increased sclerosis (Hoy et al., 2006a). Kidneys from Aboriginal people with no known kidney disease have 30% fewer glomeruli (Douglas-Denton et al., 2006; Hoy et al., 2006a) and these glomeruli tend to be larger, as determined by autopsy. It is possible that the smaller number of glomeruli is compensated by glomerulomegaly. Interestingly, the most common lesion seen in biopsies from individuals with renal disease from remote communities is glomerulomegaly and not diabetic, or other change (Hoy et al., 2014). There is a hypothesis that the smaller kidneys could be a consequence of low birth weight, a variation on Barker’s hypothesis (Manalich et al., 2000; Hoy et al., 2016). However, it is also possible that there are other factors at play.

Kidneys with fewer, large glomeruli are termed oligomeganephronic kidneys. Elsewhere in the world, this condition is most often associated with low birth weight and virtually always occurs in infants with ESRD occurring by early childhood (Wilhelm-Bals et al., 2014). A review of the literature (Alves et al., 2012) found only six cases of adult-onset oligomeganephronia. Histology on biopsies showed a pathology reminiscent of that seen in the remote Aboriginal communities with variable presence of glomerosclerosis (Alves et al., 2012). Mouse studies also suggest an inverse relationship between glomerular quantity and the presence of albuminuria (Long et al., 2013).

The Tiwi Islanders living on Bathurst and Melville Islands north of Darwin have the one of the highest measured prevalence of renal disease among Australian Aboriginals. The problem is so severe that the 3000-strong islander community requires its own dialysis unit and renal failure (start of dialysis or dying without dialysis) is the most common cause of death. It has been found that kidney disease is more common in Tiwi Islanders than three other Aboriginal communities screened and nearly 10 times that of non-Aboriginal people (OR 9.7 [8.2,11.7]) (Hoy et al., 2006b). This suggests that the Tiwi Islanders have a far greater risk of developing renal disease than other northern Australian Aboriginal communities, despite having a profile for either hypertension or diabetes similar to other communities. The Tiwi Islanders have been isolated for 15,000 years up until the last 50–100 years (Tiwi Land Council, 2018). Consequently, we hypothesize that genetic variants associated with kidney disease (in the presence of other factors brought on by quasi-western lifestyle) have become more common among the Tiwi Islanders than mainland Aboriginals. These risk alleles could have become more common with random drift, when the population was genetically isolated and the environmental risks were not present.

There are over 200 genetic changes that cause or are associated with either renal disease or renal developmental abnormalities (Vivante and Hildebrandt, 2016). While many of these are likely to be irrelevant to the Tiwi renal disease, there are several genetic changes that are associated with proteinuria alone or oligonephronia or oligomeganephronia. These include a deletion of the short arm of chromosome 4 with a residual monosomy known as Wolf–Hirschhorn syndrome where oligomeganephronia is a rare accompaniment (Park and Chi, 1993; Anderson et al., 1997). Mutations in PAX2 in both mice and humans give rise to oligomeganephronia with the occasional co-occurrence of a coloboma. And finally, hepatocyte nuclear factor-1b (HNF 1b) mutations give rise to a syndrome that includes meganephronia (Bingham et al., 2001). Current findings suggest that the excess burden of kidney disease in African Americans is due to risk alleles in the *APOL1* gene; however, these same risk alleles are not found in the Tiwi Islander community (Hoy et al., 2017).

There is evidence that renal disease in Aboriginal populations has an important genetic component. There is a significant association between a common polymorphism at codon 72 of the *p53* gene and urine albumin creatinine ratio (ACR) among a remote coastal Aboriginal Australian community in the East Arnhem region of NT (McDonald et al., 2002).

The heritability of the natural log of ACR is estimated to be 64% in an Aboriginal population, while for systolic and diastolic blood pressure, it was only 26 and 11%, respectively. The heritability of eGFR was not found to be significantly different from zero (Duffy et al., 2016). This study also displayed evidence of association of variants in *ACE* and *TP53* with ACR.

Here we describe a classical genome-wide association study (GWAS) of Tiwi Islanders in which we identified several loci associated with markers of renal disease. Two of these loci, single nucleotide polymorphisms (SNPs) rs443816 located in the gene encoding *UGT2B11* and rs4016189 located in the gene encoding *CRIM1*, were also significantly associated with the renal disease phenotype in an independently sampled cohort.

## Materials and Methods

### Sample Collection

In the 1990s, in close consultation with the Tiwi Land Council, the protocol was approved by the institutional ethics committee and written informed consent was obtained from all participants. Phenotype data were collected for 1492 samples, with sufficient DNA obtained for 249 samples.

In 2013–2014, data were collected on a second cohort of 497 Tiwi Islander study participants. This study was carried out in accordance with the recommendations of the Human Ethics Committee (Tasmania) Network ethics reference number H0012832, and in conjunction with all institutional ethics committees of the research team and the Human Research Ethics Committee of the NT Department of Health and Menzies School of Health Research. Written informed consent was obtained from all participants. Participants donated blood and urine samples, resulting in good quality DNA samples for 492 individuals. Clinical data collected include: date of birth, gender, smoking and alcohol history, blood pressure or kidney disease medicine use, height, weight, waist circumference, random blood glucose, glycated hemoglobin, serum albumin (measured by radioimmunoassay), serumcreatinine (measured by jaffe reaction), estimated glomerular filtration rate (eGFR), urinary protein, and urinary ACR. eGFR was estimated from serum creatinine levels using the MDRD Study equation (Levey et al., 1999).

In both cohorts, samples were collected on all participants who presented at the clinic and consented to be a part of the study.

### DNA Preparation and Analysis

The blood samples from which the DNA was derived for the *Affymetrix* genotyping study were collected from Tiwi Islanders during 1995–1996. Lymphocytes were obtained from buffy coat preparations, and stored under liquid nitrogen. The genomic DNA was extracted from the lymphocyte samples in 2007 using a Dneasy Blood and Tissue Kit (Qiagen). The extracted DNA was subsequently amplified using a GenomiPhi V2 DNA Amplification Kit V2 (GE Healthcare) to provide sufficient quantity and quality genomic DNA for the genotyping. For each sample, two separate 20 μL amplification reactions were conducted, and then pooled and purified using the AMPure PCR Purification System (Agencourt). The quantity and quality of the amplified and purified DNA were determined using a Nanodrop DNA Analyser (Thermo Fisher Scientific). Out of 257 lymphocyte samples subjected to DNA extraction and subsequent amplification, 251 met the minimum requirements for the genotyping analysis, namely: >2 μg DNA, 100 ng/μL, and OD260/280 ratio between 1.7–1.9.

Whole genome SNP genotyping was performed on the amplified DNA using *Affymetrix* 5.0 SNP chips. The accuracy of the genotype calls was assessed by using the following analyses. First, comparison of 25 duplicate samples indicated a concordance rate of 98.4% (range: 96.1–99.2%). Second, we used the genotype data to ascertain first- and second-degree relationships among the samples (using *PLINK* software; Purcell et al., 2007). These inferences were correctly predictive for 87% of the reported relationships. Discrepancies here are likely due to errors in self-reporting as many Tiwi Islanders are brought up by extended families and the concept of primary carer is different to other cultures.

The DNA used for the genotyping replication studies was obtained from blood samples collected from Tiwi Islanders in a separate study on the population conducted during 2015. DNA was extracted from whole blood samples using the Nucleospin Blood XL silica matrix system (Machery-Nagel) at the Australian Genome Research Facility (AGRF) in Australia. Genotyping of these samples for the SNPs identified in the GWAS stage of the study was performed using TaqMan^TM^ genotyping primers (Applied Biosystems). The SNP analysis of the *CRIM1* region was performed using the Sequenom platform (by the AGRF).

Blood samples from 12 anonymous Tiwi Islanders were sent to *Illumina* for whole genome sequencing in 2013, using the *HumanOmni2.5*-8v1 chip. The mean fold coverage for these samples ranged from 33.6 to 42.1. Given the small sample size, and the lack of phenotype information, the sequence data could not be used for association or imputation. The sequence data could be used to identify polymorphic SNPs for fine mapping.

### Population Stratification and Linkage Disequilibrium

**FIGURE 1:**
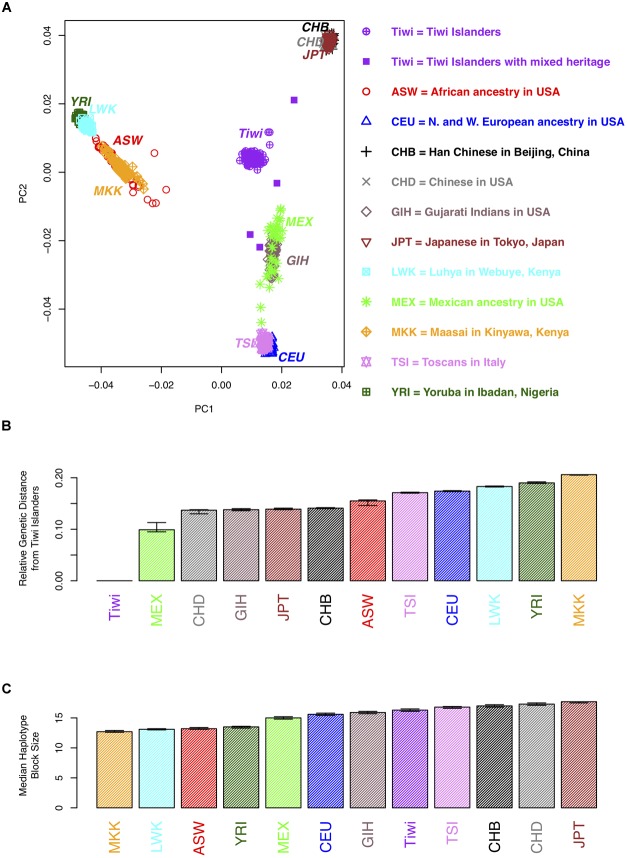
Population genetic analyses of 73 unrelated individuals from the 249 Tiwi cohort from the 1990s. **(A)** A plot of the first two principal components (PCs) for an analysis of the hapmap3 samples from 11 populations and the Tiwi cohort. Each point represents an individual. More distant points are more genetically distinct. Tiwi study participants that self-reported as mixed heritage are indicated by opaque purple squares. **(B)** The genetic distance between the midpoints of each of the hapmap3 samples and the Tiwi samples was calculated using all principal components. **(C)** The median haplotype block size of the hapmap3 samples from 11 populations and the 1990s Tiwi cohort. Haplotypes were defined using software *Haploview* based on the *four gamete rule*; 95% confidence intervals, estimated using bootstrap sampling, are displayed for the estimates in **B** and **C**.

A principal component analysis (PCA) was conducted using approximately 50,000 SNPs that were polymorphic in the Tiwi samples [minor allele frequency (MAF) > 0.2] and that were not in linkage disequilibrium (as determined using 73 unrelated Tiwi samples and *PLINK*). The PCA was performed using *EIGENSOFT* (Patterson et al., 2006) on samples from 11 populations in release 3 of phase III of the International Haplotype Map Project (hapmap3) samples. The Tiwi samples were not included in generating the principal components, to prevent the PCs from being due to differences in platforms or genotyping errors. The hapmap3 and the Tiwi samples were then projected on to a plane (Figure 1) using the first two principal components to generate a PCA plot. Eight Tiwi samples were self-reported as being of “mixed-race” and these were indicated on the PCA plot. The genetic distance between the midpoint of the Tiwi samples and the 11 hapmap3 samples was calculated using Euclidean distance including all PCs.

The median length of haplotype blocks across the genome was estimated for each of the Tiwi samples and the 11 hapmap3 samples; the median haplotype block size was then calculated across each population. The *four gamete rule* was used to define a haplotype block, dictating that all pairs of SNPs within a haplotype block exhibit less than four possible haplotypes or gametes, showing no evidence of a historical recombination within the haplotype block (Thorisson et al., 2005).

### Association Analysis

Genetic regions of homozygosity (ROH) were inferred using the “Runs of Homozygosity” option in the software *PLINK*. Homozygosity association was carried out by searching for ROH that were shared by study participants with high ACR. The data were examined to see if the high level of kidney disease could be explained through consanguinity by seeing if there was any correlation between log(ACR) and the total number and length of ROH for each individual.

For the association analysis, the natural log of ACR was used, and age and sex were included as covariates. In a previous study from the same population, heritability of ACR was estimated to be 64%, while the heritability of eGFR was not significantly greater than zero (Duffy et al., 2016). Detectable reduction in eGFR, assessed through creatinine-based measures, is a late event in the expression of renal disease in this population which is uniformly predicted by years of albuminuria, and once established progresses quickly to kidney failure. Thus, it manifests in only small numbers of people at any given study which excludes people on dialysis, and is not such a good endo-phenotype for kidney disease (Hoy et al., 2001).

The variance of ACR that can be explained by the *Affy 5.0* genotypes was examined using the *GCTA* software (Yang et al., 2011). The association analysis was conducted with a linear mixed model, also using the software *GCTA*. An additive genetic model was assumed and age was adjusted for by treating it as a covariate. The linear mixed effects regression model accounts for the non-independence among family members by modeling the variance structure of the relationships between individuals as a random effect. The analysis adjusted for relatedness by inferring it from the genome wide data. The software *GCTA* is just one of many methods for adjusting for relatedness in genome wide studies (Thomson and McWhirter, 2017). It has the advantage that we were able to obtain more power, by not including the chromosome that the locus of interest was on, in the relatedness inference (option –mlma-loco in GCTA).

To examine whether our top hit (rs4016189), which was in a gene desert, could affect transcriptional regulation of the nearest gene (*CRIM1*), we interrogated an eQTL dataset published previously (Xia et al., 2012). This dataset contains *p*-values for the association between gene expression (of all known genes) and a genome wide set of SNPs, in 11 hapmap3 samples. From this dataset, the *p*-values were extracted for association between the SNPs within the genomic region of our top hit, and regulation of *CRIM1* in the Mexican hapmap population.

### The Follow-Up Dataset

The top eight SNPs from the GWAS, were re-genotyped in the second cohort at the AGRF. These SNPs were chosen if the *p*-value of the corresponding SNP and the next closest SNP were both less than 0.0005 [corresponding to a -log _10_(p-value) greater than 3.3]. Whole genome data were not collected on all 497 Tiwi Islanders from the second cohort, so it was not possible to adjust for relatedness. A simple linear regression model was used to measure the significance of association for the top eight SNPs and the fine mapping analyses. The covariate, age was adjusted for, and an additive genetic model was assumed.

### Structural Equation Model

A structural equation model (SEM) was used to examine the genotype most associated with ACR, and its effect on other phenotypes. SEMs provide an approach to propose a causal hypothesis in a statistical framework that can then yield causal interpretations conditional on the model assumptions and data. The *R* package; *piecewiseSEM* was used to estimate the coefficients that describe the strength of the causal pathways in the SEM, as it allowed the use of generalized linear models (Lefcheck, 2016). Dependent variables in the SEM are ACR, eGFR, systolic blood pressure (sysBP), and diabetes (HbA1c > 6.5). ACR was logged and diabetes is modeled using a logistic regression model to match the assumptions of regression. The SEM was modeled assuming a causal relationship from diabetes to blood pressure and to ACR/eGFR and that ACR and eGFR are correlated. Using the standardized coefficients, the effect sizes of the genotype (rs4016189), age, sex, and body mass index (BMI) on the dependent variables were compared.

## Results

### Phenotypes

The summary statistics of kidney disease, blood pressure, diabetes, age, and BMI are shown in Table 1 for the two cohorts.

**Table 1:** Mean/median/proportion of the variables of interest in the two cohorts of Tiwi study participants.

	1990s Cohort	2013–4 Cohort
Number of participants with genotype data	249	497
Age (mean, range) (years)	33.3 (12–73)	39.7 (17–75)
Sex (number of males/females)	133 M, 116 F	253 M, 239 F
ACR (median, IQR, range) (mg/g creat)	20 (7–249) (1–6058)	23 (7–159) (1–17,555)
eGFR (mean, range) (mls/min/1.73 m^2^)	105.9 (25–243)	84.4 (5–90)
Systolic BP (mean, range) (mmHg)	121.2 (84–170)	114.5 (71–187)
Diastolic BP (mean, range) (mmHg)	73.2 (39–122)	73.8 (48–124)
Diabetes (Y/N in 1990s cohort^∗^, hba1c > 6.5 in 2013–4 cohort)	8.5%	18.2%
BMI (median and IQR)	22.3 (19–27)	23.8 (21–28)
Relationships	135 (0.5%) are first degree relationships, 307 (1.2%) are second degree relationships.	Unknown

*^∗^In the 1990s cohort, a study participant was deemed to have diabetes if a clinical history was in the records, or if they were on hypoglycemic medications, or WHO diagnostic levels of glycemia fasting or were 2 h after oral glucose challenge.*

The variance in ACR, explained by genotype as measured by the *Affymetrix* 370K SNP chips was 37% (estimated using the software GCTA), after adjusting for age and sex. This was significantly greater than zero (likelihood ratio = 8.06, *p*-value = 0.002), showing that the genetic variation in the SNPs genotyped has a significant effect on the risk of kidney disease, and the SNPs explained 37% of the variation in ACR among the Tiwi Islanders. We would expect that the variance explained by the whole genome sequence data to be closer to the heritability estimate of 64% (Duffy et al., 2016), as there are likely to be rare variants that also contribute to variation in ACR.

### Genotypes

The *Affymetrix* 5.0 SNP chip is made up of approximately 500,000 SNPs that are known to have a MAF > 0.05 in a Caucasian population. Based on the 249 individuals that were part of the 1990s sample, for the Tiwi population, 40% of these SNPs have a MAF < 0.05 and 35,000 of these SNPs are monomorphic. This high rate of monomorphism may be because *Affymetrix* 5.0 SNPs were selected to be polymorphic in a Caucasian population, while other SNPs could be polymorphic in the Tiwi population.

We used the genome wide SNP data to conduct a PCA to compare the Tiwi samples with several other ethnic groups and to distinguish whether there was any evidence of population stratification within the Tiwi samples. The PCA results (Figure 1A,B and Supplementary Table S1) distinguish several different ethnic groups into individual clusters, including the African Masssai, Luhya and Yoruba people, and Mexicans. The Han and United States-based Chinese and Japanese groups clustered together in a separate domain, as did the Tuscans and CEPH European ancestry groups. Strikingly, the Tiwi clustered together in another completely separate domain of the PCA plot. Eight Tiwi samples were self-reported as being of “mixed-race.” Four of these were noticeably separated from the other Tiwi samples. However, there was no evidence of population stratification within the Tiwi samples.

The median haplotype block size of the Tiwi samples was 16.3 kb (95% C.I.: 16.1–16.5 kb), based on the *four gamete rule*. When comparing this estimate to the median haplotype block size to the other populations, LD extends further in the Tiwi samples than the African and some other populations (Figure 1C and Supplementary Table S1). However, LD does not extend further than the Chinese, Japanese, or Tuscan populations, suggesting that the Tiwi population have a distant common ancestor, despite their small effective population size.

### Association

Genetic ROH were inferred using the “Runs of Homozygosity” option in the software *PLINK*. We found that there were no runs of inferred homozygosity that were shared by more than two individuals. The total length of inferred ROH was not significantly longer for individuals with higher ACR (*kendall’s r* = 0.018, *p*-value = 0.67), suggesting that higher levels homozygosity do not explain the higher levels of kidney disease in the Tiwi population. There were very few ROH inferred that were less than 2 Mb in length. This may be due to the high genotyping error rate or low coverage. Consequently, it may be possible that the higher levels of kidney disease in the Tiwi population could be due, in part, to shorter runs of homozygosity that are less than 2 Mb in length.

**FIGURE 2:**
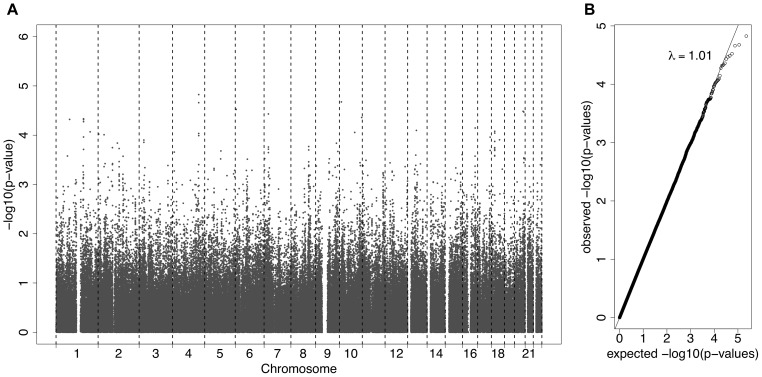
**(A)** A Manhattan plot displaying the –log10(*p*-values) for the association between the phenotype ACR and the SNPs from the *Affymetrix* SNP Chip. **(B)** A QQ-plot, showing the observed *p*-values on the *y*-axis and the expected *p*-values on the *x*-axis under the null hypothesis of no association.

A GWAS was carried out using GCTA analysis, adjusting for age and sex, with log(ACR) as the phenotype. This is a mixed effects analysis that accounts for relatedness. Figure 2A presents all the association results as -log10(*p*-values) across the genome as a Manhattan plot. A quantile-quantile plot (Figure 2B) displays the expected vs observed *p*-values as points on a scatter plot, ordered from largest to smallest. The majority of the points on the scatterplot lie on the one-to-one line, suggesting that the *p*-values are not over-inflated (as could happen with unaccounted relatedness or population stratification). The most significant SNPs (largest -log_10_ *p*-values) were not as significant as expected, suggesting that the study may be underpowered, necessitating replication.

**FIGURE 3:**
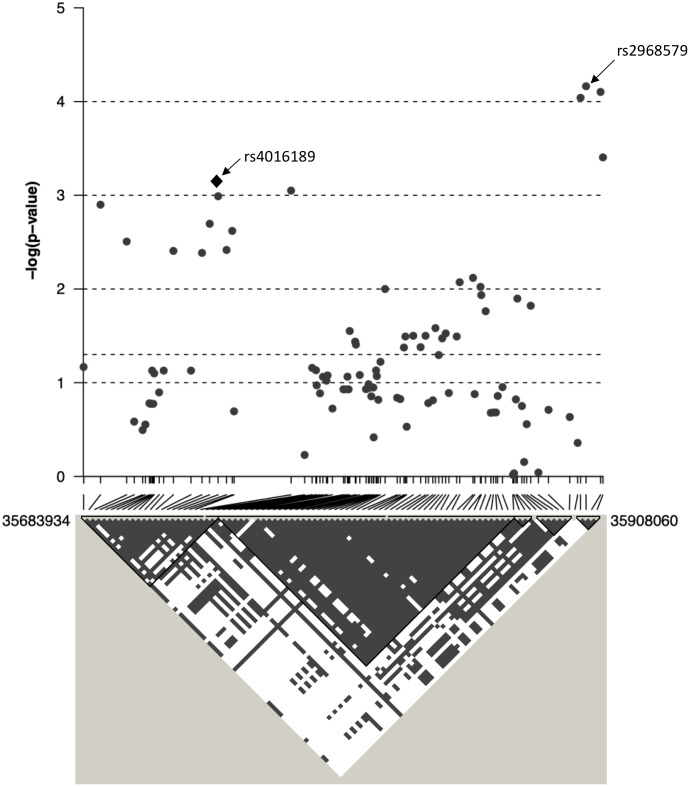
The association between ACR and 97 SNPs in the region surrounding rs4016189 is displayed as –log10 *p*-values. CRIM1 gene lies 448 kb upstream of the region displayed on this graph. Positions are based on ensembl GRCh38.p12. Below the graph, the linkage disequilibrium between each of the 97 SNPs is displayed as gray/white squares, using the *four gamete rule*. SNPs that do not show all four possible haplotypes/gametes (i.e., in LD) are colored gray (as opposed to white).

The top eight SNPs were chosen for follow up in an independent sample (Table 2). These SNPs were chosen when the *p*-value of the corresponding SNP and the next closest SNP were both less than 0.0005 [corresponding to a -log _10_(p-value) greater than 3.3]. Among the top hits identified in the GWAS were SNPs located in or near genes with potential roles affecting kidney function and development. The SNP with the lowest significance, rs4016189 (*p*-value = 9.76 × 10^-5^), was located in a large intergenic region and approximately 614 kb upstream of the *CRIM1* gene on chromosome 2p2.22; the next closest protein-coding gene to this SNP was more than 1 Mb distant (in the opposite direction to *CRIM1*). The association between this variant and ACR levels also remained highly significant in the replication study (*p*-value = 0.000751). The associated allele was more common in both Tiwi cohorts, than other populations (Table 3). *CRIM1* encodes Cysteine Rich Transmembrane BMP Regulator 1, a transmembrane protein containing six cysteine-rich repeat domains and an insulin-like growth factor-binding domain. It is known to have roles in renal development (Wilkinson et al., 2012; Phua et al., 2013) and mice with a *Crim1* knockdown develop a glomerulomegaly (Georgas et al., 2000) and therefore was of high interest to our study. To test the possibility that the SNP could be involved in transcriptional regulation of *CRIM1*, we interrogated the genomic region containing this SNP in an eQTL dataset published previously (Xia et al., 2012). We observed a significant association between several variants, including rs4016189, and *CRIM1* expression in the hapmap3 MEX population (Supplementary Figure S1). We also conducted a fine scale genetic analysis of the region near rs4016189 and the *CRIM1* locus. To do this, we identified variants spanning approximately every 2 kb within the region (using whole genome sequences from 12 Tiwi individuals), developed Sequenom assays for each variant and genotyped these in the 497 samples in the 2013–14 cohort. The results of the association tests conducted for each of these SNPs (97 in total) with ACR levels are presented in Figure 3. As well as confirming the rs4016189 association using this independent assay, we observed multiple SNPs in this region, including those located nearest *CRIM1* (e.g., rs2968579; 455 kb away from the gene) were also significantly associated. This provided additional evidence that a region upstream and near to *CRIM1* was associated ACR levels. Figure 3 also displays the LD between these 97 SNPs. Three distinct haplotype blocks can be seen across the region, although there is some evidence that the SNPs around rs4016189 are in LD with the SNPs around rs2968579.

**Table 2:** The top eight hits in the GWAS study on the original cohort, along with the association results in the replication cohort.

Chr	SNP	Position (bp) GRCh38.p12	Assoc. allele	Nearest gene	*p*-value in original sample	*p*-value of nearest SNP in original sample	*p*-value in replication sample
2	rs4016189	35741807	C	CRIM1 (614 kbp away)	9.76E-05	0.000433	0.000751
4	rs4438816	69165940	T	UGT2B11	0.000739	5.24E-05	0.022
4	rs6823947	69545677	A	UGT2B11	5.24E-05	0.000294	0.079
4	rs12511454	69566288	G	UGT2B11	0.000294	5.24E-05	0.078
7	rs6461901	25990123	A	NFE2L3 (162 kb away)	3.70E-05	0.000173	0.035
9	rs1535656	121188709	C	RAB14	0.000144	0.000145	0.037
17	rs2279057	76313344	C	PRPSAP1	0.000109	0.000203	0.069
20	rs2254239	56994842	C	lncRNA, LOC105372683 (8 kb away)	3.39E-05	0.000288	0.962

**Table 3:** Allele frequencies of the associated alleles for SNPs that were significant in the replication study (p < 0.05).

Chr	SNP	Assoc allele	Gene	Allele freq. in Tiwi Cohort 1	Allele freq. in Tiwi Cohort 2	Allele freq in MEX	Allele freq. in YRI	in CEU
2	rs4016189	C	CRIM1 (614 kbp away)	0.83	0.82	0.36	0.55	0.52
4	rs4438816	T	UGT2B11	0.40	0.46	0.25	0.26	0.18
7	rs6461901	A	NFE2L3 (162 kb away)	0.21	0.25	0.08	0.75	0.14
9	rs1535656	C	RAB14	0.27	0.24	0.26	0.01	0.10

*Allele frequencies for the other populations were obtained from https://www.ncbi.nlm.nih.gov/variation/tools/1000genomes/. MEX = Mexican Ancestry in the United States, YRI = Yoruba in Ibadan, Nigeria, CEU = North and Western European Ancestry in United States.*

Three other SNPs, rs4438816, rs6823947, and rs12511454 associated with ACR in the GWAS were located within the same gene, *UGT2B11* on chromosome 4q13.2. The gene encodes the enzyme UDP Glucuronosyltransferase (UGT) Family 2 Member B11 (EC 2.4.1.17) – none of the associated SNPs changed the coding sequence of this gene. The former SNP was also significantly associated with ACR in the replication study (*p* = 0.022), although associations for the other two did not quite reach significance (*p* = 0.079 and 0.078). The associated allele for rs4438816 was more common in both Tiwi cohorts, than other populations (Table 3). Members of the UGT enzyme family catalyze an intracellular process known as glucuronidation, in which potentially toxic endogenous compounds as well as many drugs and xenobiotics are conjugated and subsequently eliminated from the body. Previous studies have shown that some xenobiotics cause kidney disease, such as Balkan endemic nephropathy (BEN) caused by exposure to aristolochic acid present in flour produced from wheat from the endemic areas (Stefanovic and Polenakovic, 2009).

Two additional SNPs, rs6461901 and rs1535656, were also significantly associated with ACR levels in the replication study (*p* < 0.05). The associated alleles in these two SNPs did not consistently have higher allele frequencies in the Tiwi samples, compared to other populations (Table 3). The former SNP is located in an intergenic region and approximately 162 kb upstream of *NFE2L3* on chromosome 2q31.2. *NFE2L3* encodes a transcription factor, nuclear factor erythroid 2-related factor 2 (NRF2), which has been widely studied for its cytoprotective functions, including in kidney disease (Nezu et al., 2017). The other SNP was located in an intronic region of *RAB14* on chromosome 9p33.2. *RAB14* encodes a GTPase expressed in many cell types, including the kidney, and is involved the regulation of ER protein trafficking (Junutula et al., 2004). No obvious roles in kidney function or development have been ascribed to this gene. None of the other SNP identified in the GWAS were significantly associated with ACR levels in the replication study.

### Associations With Other Phenotypes

**FIGURE 4:**
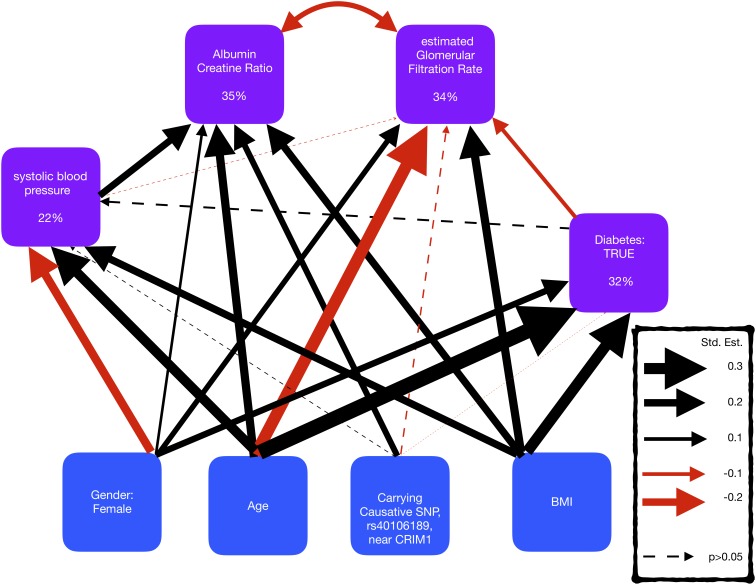
Representation of a structural equation model, exploring the comorbidity of kidney disease, blood pressure, and diabetes. The boxes represent the measures and the arrows show the relationships between the variables (black/red arrows for positive/negative relationships, respectively). The thickness of the arrows has been scaled based on the magnitude of standardized regression coefficients for putative causative relationships (straight arrows) and correlation coefficients for correlated variables (curved arrows). The line type of the arrow denotes significance (solid = >*p*-value < 0.05; dotted => *p*-value > 0.05). The percentage variance explained by the risk factors is also shown in the boxes. The models also adjusted for cohort, but coefficients are not shown here to make the graphs simpler.

A SEM was fitted to compare the effect sizes of the SNP rs4016189, that is near CRIM1, with known risk factors for kidney disease, blood pressure, and diabetes (Figure 4 and Supplementary Table S2). The SEM consisted of four regression equations, explaining the variation in four dependent variables; ACR, eGFR, sysBP, and diabetes (HbA1c > 6.5). ACR and eGFR were negatively correlated (red curved arrow) as expected, since high ACR and low eGFR correspond to kidney disease.

The SEM displayed that the SNP, rs4016189 was significantly independently associated with ACR, but not with sysBP, diabetes, or eGFR. The effect size of this SNP on ACR was smaller than age, BMI, and sysBP, but larger than gender.

The largest standardized effect size in the SEM was the positive effect of age on diabetes, followed by the negative effect of age on eGFR. The risk factors described the largest proportion of the variation in ACR, and the smallest variation in sysBP, suggesting that there are other risk factors involved in sysBP.

## Discussion

Australian Aboriginals experience a high burden of kidney disease that is at least partly independent of other comorbidities that affect kidney function. The problem is particularly acute among the Tiwi Islanders, where rates of ESKD are 30 times that of the general Australian population. The Tiwi have lived in relative isolation for probably thousands of years, and anecdotally, did not suffer from renal disease prior to adopting a quasi-Western lifestyle sometime during the mid-late 20th century. It is possible that dietary and/or other environmental factors introduced since this time have contributed to the rapid emergence of the disease. However, our previous studies indicated a significant heritable component (Duffy et al., 2016), raising the possibility that individuals are genetically predisposed to the disease, but only in the presence of these hypothetical environmental risk factors, i.e., gene–environment interactions. In this study, we investigated the genetic contribution to renal disease in Tiwi Islanders by conducting a GWAS, in which associations were tested between SNP genetic variants and single measure ACR levels. A number of nominally significantly associated SNPs were identified. These SNPs did not reach genome wide significance, probably due to the small sample size. The top eight SNPs were re-tested for association in a separately collected cohort from the same population. Four of these SNPs were significantly associated with ACR in the replication sample (*p* < 0.05), and through examination of their known biology and functions, there are possible mechanisms through which renal disease in Tiwi Islanders may manifest.

We identified a genomic region upstream and near to the *CRIM1* locus. The prototypical GWAS SNP in this region, rs4016189, was also significantly associated in the replication study, as were a set of additional SNPs located between this SNP and *CRIM1*. The indicative region of association spans at least 250 kb; however, our analysis did not extend sufficiently to include the *CRIM1* locus itself. The associated region may be involved in regulating *CRIM1* transcription, as suggested by the positive eQTL signal from the rs4016189 SNP. It is also possible that there are other more distantly located variants that are in linkage disequilibrium that influence gene function, although we did not observe any non-synonymous changes in *CRIM1* in the 12 genome-sequenced samples. A further in-depth study of the interval and the relationship between sequence variation and *CRIM1* gene expression will be required to understand the functional link between the associated variant and disease phenotype. The function of *CRIM1* is not well understood, but it has several important roles in development, including in the kidney. Basic studies in the mouse demonstrate that the murine homolog, *Crim1*, is expressed in the developing kidney (Georgas et al., 2000); it is also localized in descending loop of Henle cells in the adult kidney (Park et al., 2018). In humans, immunohistochemical studies of have localized CRIM1 to the podocyte slit diaphragm of the adult human kidney (Nystrom et al., 2009). *Crim1* knockout mice die *in utero* from multiple organ defects. However, a subset of mice homozygous for a *Crim1* hypomorphic mutation, called KTS264 (Phua et al., 2013) survive to adulthood and go on to develop a glomerulomegaly that is reminiscent of that seen in biopsy studies of Australian Aboriginal renal patients. It is therefore tempting to speculate that the renal disease associated variants modify *CRIM1* expression in such a way as to affect the developing kidney and lead, possibly in conjunction with added environmental factor(s), to the renal disease susceptibility phenotype in the Tiwi. That the associated variant may operate directly through effects on kidney function is further supported by our structural equation modeling, which showed no relationships between the variant and kidney disease risk factors such as diabetes and sysBP. However, age, BMI, and blood pressure were all shown to have a greater effect on urinary ACR than the prototypical SNP. Therefore, the underlying causes of disease are clearly complex.

The GWAS also implicated a locus encoding a UGT, *UGT2B11*, in the renal disease susceptibility. Three SNPs in this gene were independently associated with ACR, although only one remained significant in the replication study. The functions of UTGs, and their known involvement in disease, indicate their possible involvement in renal disease pathophysiology. UGTs catalyze the conjugation of glucuronide moieties to many drugs and xenobiotic compounds, an important step in the Phase II detoxification pathway of drug metabolism. Exposure to certain xenobiotics has been shown to cause renal disease. For example, BEN which results from exposure to aristolochic acid present in flour produced in this region (Arsenović et al., 2005) and in animals exposed to mycotoxins (fungal toxins) in contaminated feedstock (Austwick, 1984). Interestingly, BEN is only observed in some households despite the ubiquitous exposure to this xenobiotic, and both a genetic predisposition and environmental (xenobiotic) influence have therefore been implicated. A potential role for genetic factors influencing xenobiotic metabolism is exemplified in a common polymorphism causing UGT2B17 deficiency. UGT2B17 is known to conjugate testosterone and other androgens, and thus enable their excretion in the urine. The genetic deficiency causes dysregulation of testosterone levels including reduced urinary excretion (Jakobsson et al., 2006). In androgen anabolic steroid (AAS) use, urinary detection of the drug is compromised in UGT2B17 deficient individuals and affected abusers can develop renal function problems, presumably because of build-up of AAS (Deshmukh et al., 2010). Therefore, we hypothesize that the renal disease associated UGT2B11 variant identified in the Tiwi may compromise an ability to conjugate and thus excrete a nephrotoxic xenobiotic or produce a nephrotoxic xenobiotic by conjugation of pro-substance. It is also conceivable that the introduction of this compound coincided with their recent adoption of a Westernized life-style and changed living conditions.

A third associated SNP, rs6461901, lies near a gene whose product, NRF2, is a well-characterized transcriptional regulator that directs the expression of cellular systems employed to protect against oxidant and electrophile-induced stress. Oxidative damage is central to organ damage following ischemic injury, for example, and in the kidney upregulation of NRF2 function provides protection against the development of chronic kidney disease resulting from such injury. This has been shown in many studied in mice with genetic modifications affecting NRF2 function and in human clinical trials using small molecule activators of NRF2 (reviewed in Nezu et al., 2017). We therefore speculate that altered NRF2 expression or function could affect the severity of renal disease in the Tiwi, although we have no knowledge of how the associated variant may affect the gene or its product.

The Tiwi people have been proactively engaged with research in their community for 30 years or more. In the 1980s, research was seen as exploitative by many Indigenous leaders; at that time when there was no national leadership to provide ethical guidance for researchers and support for communities (Kowal and Anderson, 2012). The Tiwi people worked with the Menzies School of Health Research from its earliest years; Menzies had established an ethics committee with Indigenous members to oversight its research. Subsequently, the Tiwi Land Council, through its Health Board, signed an historic research agreement with Menzies to formalize Tiwi control of the research priorities, research information, and samples. This is believed to have been the first ever such legal agreement between an Indigenous community and a research organization.

The Menzies School of Health Research had also helped to focus the national debate by organizing the 1986 conference in Alice Springs, attended by Indigenous leaders, researchers, and NHMRC representatives; this helped to launch a process that eventually led to NHMRC research guidelines to support community decision-making and control of Indigenous health research.

The Tiwi people’s engagement with research has had a particular emphasis on chronic disease and kidney disease. They have articulated and shaped the research directions, raised local financial support and raised external funds, specifically the Stanley Tipiloura Fund (to honor the first Tiwi MLA) to support the first phases of this research, worked with government agencies, including Australia’s National Health and Medical Research Council, with Kidney Health Australia (and through them, Rio Tinto), and with Servier, Australia. Tiwi people have worked as staff and leaders in all the research projects conducted within their community, and Tiwi people have participated in many surveys, treatment programs, and controlled clinical trials. Tiwi people pioneered governance of research processes with formation of the Tiwi Health Board, founded their own Scientific Research and Advisory Group and Committee. Tiwi advocated for and house the first satellite dialysis unit in the NT. Tiwi people encouraged application of genetics research, to illuminate their origins, migrations, customs, relationships, and health issues, and they are proud to be contributors on the world stage.

We also investigated the relative degree of genetic relatedness between the Tiwi and other populations using PCA and a set of polymorphic SNPs identified in the Tiwi GWAS study. We observed that the Tiwi samples clustered in a single group, that was well-separated and distant from all of the other populations tested. As an example of the relative genetic differences observed, the approximate genetic distance separating the closest population to the Tiwi, Mexicans, was similar to that separating Mexicans from Europeans. Collectively, the analysis is strongly suggestive of the Tiwi’s long existence as a genetically distinct and isolated population. These data have been discussed with members of the Tiwi Land Council and has elicited a large degree of interest, as it is pertinent to the story of the origins of the Tiwi.

## Ethics Statement

In the 1990s, in close consultation with the Tiwi Land Council, the protocol was approved by the institutional ethics committee and written informed consent was obtained from all participants. In 2013–2014, data were collected on a second cohort of 497 Tiwi Islander study participants. This study was carried out in accordance with the recommendations of the Human Ethics Committee (Tasmania) Network ethics reference number H0012832, and in conjunction with all institutional ethics committees of the research team and the Human Research Ethics Committee of the Northern Territory Department of Health and Menzies School of Health Research. Written informed consent was obtained from all participants.

## Author Contributions

RT carried out the statistical analyses and wrote the manuscript. TT contributed to the statistical analyses. WH, JM, and SF conceived the study. MJ contributed clinical knowledge. BM and GB carried out biochemical analyses and genotyping. LW carried out the DNA amplification. All authors read and approved the final manuscript.

## Conflict of Interest Statement

The authors declare that the research was conducted in the absence of any commercial or financial relationships that could be construed as a potential conflict of interest.
