# Supplementary material for: New Genetic Loci Associated With Chronic Kidney Disease in an Indigenous Australian Population
Source: Front Genet. 2019 Apr 16;10:330. doi: 10.3389/fgene.2019.00330 (PMC6476903; doi:10.3389/fgene.2019.00330)
Supplement: Supplementary file 1 [file Table_1.DOCX]

**Supplementary Material**

| Population | Genetic Distance to Tiwi Population (bootstrapped 95% C.I.) | Median Haplotype Block Size (95% CI of the Median) |
| --- | --- | --- |
| Tiwi | 0 | 16.3kb (16.1-16.5) |
| Mexican ancestry in USA | 0.099 (0.095,0.113) | 15kb (14.8-15.2) |
| Chinese in USA | 0.137 (0.13,0.138) | 17.3kb (17.1-17.5) |
| Gujarati Indians in USA | 0.138 (0.136,0.14) | 15.9kb (15.7-16.1) |
| Japanese in Tokyo, Japan | 0.139 (0.138,0.141) | 17.7kb (17.5-17.9) |
| Han Chinese in Beijing, China | 0.141 (0.14,0.142) | 17kb (16.8-17.2) |
| African ancestry in USA | 0.155 (0.146,0.157) | 13.2kb (13.1-13.4) |
| Tuscans in Italy | 0.171 (0.17,0.172) | 16.8kb (16.6-16.9) |
| N. and W. European ancestry in USA | 0.174 (0.173,0.175) | 15.6kb (15.4-15.8) |
| Luhya in Webuye, Kenya | 0.183 (0.182,0.184) | 13.1kb (13-13.2) |
| Yoruba in Ibadan, Nigeria | 0.19 (0.189,0.192) | 13.5kb (13.3-13.6) |
| Maasai in Kinyawa, Kenya | 0.206 (0.205,0.209) | 12.7kb (12.6-12.9) |

Supplementary Table 1. Genetic Distance to Tiwi Samples and medium haplotype block size for a number of populations. Haplotype blocks are defined using the *four gamete rule*. Calculations for the Tiwi populations were based on 73 unrelated individuals.

| Response | Predictor | Estimate | Std.Error | Crit.Value | P.Value | Std.Estimate |
| --- | --- | --- | --- | --- | --- | --- |
| ACR | age | 0.0371 | 0.0062 | 5.9843 | 0 | 0.2226 |
| ACR | sex=F | 0.296 | 0.1484 | 1.9947 | 0.0465 | 0.0709 |
| ACR | cohort=2014 | -0.4632 | 0.1622 | -2.8553 | 0.0045 | -0.103 |
| ACR | systolic BP | 0.0246 | 0.0051 | 4.8557 | 0 | 0.1848 |
| ACR | BMI | 0.0701 | 0.0129 | 5.441 | 0 | 0.1979 |
| ACR | Diabetic | 1.4036 | 0.2144 | 6.5465 | 0 | 0.2451 |
| ACR | rs4016189 | 0.6263 | 0.1304 | 4.8042 | 0 | 0.1621 |
| eGFR | age | -0.6107 | 0.0702 | -8.7046 | 0 | -0.3249 |
| eGFR | sex=F | 6.8102 | 1.6809 | 4.0516 | 1.00E-04 | 0.1446 |
| eGFR | cohort=2014 | -18.0694 | 1.8375 | -9.8338 | 0 | -0.3559 |
| eGFR | systolic BP | -0.0158 | 0.0574 | -0.2745 | 0.7838 | -0.0105 |
| eGFR | BMI | 0.8881 | 0.1459 | 6.0868 | 0 | 0.2222 |
| eGFR | Diabetic | -6.8598 | 2.4285 | -2.8247 | 0.0049 | -0.1061 |
| eGFR | rs4016189 | -1.7148 | 1.4766 | -1.1613 | 0.246 | -0.0393 |
| systolic BP | age | 0.3307 | 0.049 | 6.7481 | 0 | 0.2644 |
| systolic BP | sex=F | -7.0957 | 1.1834 | -5.9962 | 0 | -0.2264 |
| systolic BP | cohort=2014 | -9.7109 | 1.2705 | -7.6433 | 0 | -0.2874 |
| systolic BP | BMI | 0.5076 | 0.1037 | 4.8933 | 0 | 0.1908 |
| systolic BP | Diabetic | 2.7617 | 1.7585 | 1.5705 | 0.1168 | 0.0642 |
| systolic BP | rs4016189 | 0.5803 | 1.0712 | 0.5417 | 0.5882 | 0.02 |
| Diabetic | age | 0.0756 | 0.0114 | 6.6253 | 0 | 0.4016 |
| Diabetic | sex=F | 0.8131 | 0.2673 | 3.0416 | 0.0024 | 0.1723 |
| Diabetic | cohort=2014 | 0.9838 | 0.3416 | 2.8801 | 0.004 | 0.1934 |
| Diabetic | BMI | 0.1185 | 0.0209 | 5.6625 | 0 | 0.296 |
| Diabetic | rs4016189 | -0.0076 | 0.2393 | -0.0317 | 0.9747 | -0.0017 |
| GFR | ACR | -0.1415 |  | -3.4436 | 3.00E-04 | -0.1415 |

Supplementary Table 2. Results from Structural Equation Model that are displayed in Figure 4.


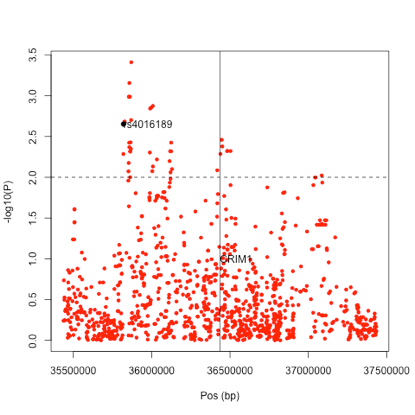


Supplementary Figure 1: Association between SNPs and expression of the CRIM1 gene, using the MEX hapmap data. Data obtained online from eQTL database, *seeQTL*.
